# Occult Infection in Aseptic Joint Loosening and the Diagnostic Role of Implant Sonication

**DOI:** 10.1155/2015/946215

**Published:** 2015-10-25

**Authors:** J. T. Kempthorne, R. Ailabouni, S. Raniga, D. Hammer, G. Hooper

**Affiliations:** ^1^Department of Orthopaedic Surgery, Christchurch Hospital, Private Bag 4710, Christchurch 8140, New Zealand; ^2^Department of Microbiology, Northland DHB, Private Bag 9742, Whangarei 0148, New Zealand; ^3^Department of Orthopaedic Surgery and Musculoskeletal Medicine, University of Otago, Christchurch 8140, New Zealand

## Abstract

Our aim was to determine the incidence of occult infection and to examine the role of ultrasound sonication of the implants in cases of presumed aseptic loosening in a prospective trial. Joint swabs, aspirates, and deep tissue samples were obtained from around the prosthesis for routine microbiology. Each prosthesis was sonicated and the sonicate examined with Gram staining and extended cultures. There were 106 joints in the study of which 54 were revised for aseptic loosening and 52 were assigned to the control revision group. There were 9 positive cultures with 8/54 positive cultures in the aseptic loosening group and 1/52 in the control revision group (*p* = 0.017, associated OR 47.7). We found concordant results between sonication fluid culture and conventional samples in 5/9 cultures. Preoperative inflammatory markers were not prognostic for infection.* Coagulase-negative Staphylococcus *was the most commonly cultured organism (7/9). Previously unrecognised infection was present in 15% of patients undergoing revision for aseptic loosening. Ultrasound sonication of the removed prosthesis was less sensitive than conventional sampling techniques. We recommend routine intraoperative sampling for patients having revision for aseptic loosening, but we do not support the routine use of ultrasound sonication for its detection.

## 1. Introduction

New Zealand has an increasing aging active population with increasing rates of primary and revision joint arthroplasty surgery. In 2009, 1467 hip and knee revision arthroplasty operations were conducted nationwide, which represented 11% of all hip and knee arthroplasty operations performed during that time period. Aseptic loosening was one of the most common indications for revision, being the cause for revision in 35% and 23% of total hip and knee joints, respectively [1]. Differentiating between prosthetic joint infection (PJI) and aseptic loosening is important, as the treatment of the two groups is significantly different. Despite attempts to differentiate between the two, no single investigation has been identified as being reliable in determining the presence of infection.

The American Academy of Orthopaedic Surgeons guidelines for the diagnosis of PJI of the hip and knee recommend risk stratification on the basis of preoperative C-reactive protein and erythrocyte sedimentation rates and selective hip aspiration in patients where infection is suspected or likely [2]. On the basis of current evidence, they do not recommend intraoperative Gram stain, but they do recommend intraoperative frozen section and cultures in cases of likely infection.

Some microorganisms, especially* Staphylococcus *species, are able to attach to implants in a glycocalyx biofilm [3, 4]. These biofilms make the identification and treatment of the microorganism more difficult. Unrecognized or occult infection has been implicated in contributing to “aseptic” loosening of joint prostheses [3, 5, 6]. Recent literature has suggested that sonication (ultrasonic cleaning submersed in a solution) and culture of extracted implants may be a reliable method of improving the sensitivity in the detection of PJI and may be superior to standard tissue cultures [6–10].

The first aim of this study was to determine the prevalence of occult or unrecognized infection in a local sample of patients undergoing joint revision surgery for presumed aseptic loosening. The second aim was to determine whether ultrasound sonication of the removed implants improved the detection of microorganisms over conventional sampling methods, in patients with low risk preoperative risk of deep infection. Our hypothesis was that sonication and subsequent culture of retrieved implants would be more sensitive at detecting occult infection than conventional culture techniques in cases of presumed aseptic loosening.

## 2. Methods

Prior to the initiation of the trial, we undertook quality control studies to ensure that we did not have false culture growths secondary to contamination or false negative results due to organism destruction. First we used 15 sterilized and unused joint prostheses that were removed directly from their packaging and processed through the sonication protocol, adhering to sterile surgical technique. There were no positive culture results from these implants. The effect of the study's sonication protocol on microorganism viability was also assessed in a series of* in vitro* experiments where the colony counts were compared between sonicated and nonsonicated* Staphylococcus aureus* and* Escherichia coli *standardized colonies. There were no differences between the two groups, which confirmed that there were no detrimental effects of the sonication protocol on both Gram positive and Gram negative microorganism viability.

We undertook a prospective case-control study, from 2008 until 2011, at two tertiary referral hospitals in Christchurch, New Zealand. Participation in the study was dependent on surgeons' and patient's wishes. Verbal and written consent was obtained from the patient by their orthopaedic consultant surgeon or registrar. The only exclusion criteria were a refusal by a patient to participate in the study or a failure to adhere to the study protocol. The study design was assessed and approved by the Upper South Island Regional Ethics Committee (URA/08/03/EXP).

The indication for revision surgery was used to divide the patients into two groups. The first was an aseptic loosening (study) group. These patients had a loose prosthesis and a clinically determined unlikely infection based on routine preoperative investigations. Cases were defined as infected if there were any of the following: (1) presence of a sinus tract to the joint, (2) a positive culture sample from the affected joint preoperatively, (3) an acutely unwell patient with elevated inflammatory markers, and (4) gross purulence at the time of revision surgery. The second was a control revision group, which included patients undergoing revision surgery for isolated polyethylene wear with liner exchange, dislocation, periprosthetic fracture, and mechanical failure of an implant.

All patients had preoperative blood tests that included a full blood count with white cell differential. We had incomplete acquisition of erythrocyte sedimentation rate (ESR) and C-reactive protein (CRP) for the patients in the study. Preoperative joint aspirates were not routinely performed unless there was clinical concern for acute or low grade infection. Prophylactic antibiotics were administered as per the surgeons' wishes, as a requirement for ethical approval, and therefore some patients received these at induction.

At the time of revision surgery, once the joint capsule was entered, one swab, a joint fluid aspirate, and several deep tissue samples from around the prosthesis were obtained. Any presence of frank purulence or lack of it was documented. The samples were sent to the laboratory for gram staining and conventional culture on chocolate and sheep blood agar. Once a prosthetic component was extracted, any gross contamination with tissue, bone fragments, and/or cement was gently cleaned off by rinsing the implant in a bowl of sterile saline. Handling of the implant was minimized. It was then placed into a sterile leak proof plastic container by the surgical team. The implant was fully submersed in sterile saline and then the container closed and sealed with adhesive tape. This was done to eliminate leakage and contamination in transit to the laboratory where all the processing of the removed prostheses occurred.

Each prosthesis was sonicated, in the original surgical container, for 10 minutes at 67 kHz using a Cavitator Ultrasonic Cleaner (Mettler Electronics, Anaheim, CA, USA). After sonication, a 50 mL aliquot of the sonicated fluid was centrifuged at 3000 rpm for 15 minutes. The supernatant was discarded and the pellet was cultured aerobically on blood and chocolate agar and anaerobically on blood agar. A gram stain alcohol washed slide was then prepared for review and extended cultures performed for 14 days.

A review of the patient's electronic records was used to collect data on patient demographics and historical details of the joint undergoing revision and data on the following risk factors: smoking status, history of diabetes mellitus, prolonged steroid use (more than 6 months), and obesity (BMI >40). The preoperative radiographs were reviewed to determine whether the implants were uncemented, hybrid, or cemented joints.

Statistical analysis was performed using a PASW SPSS 18.0.0 Statistical Software package (IBM, Chicago, IL, USA). Pearson's Chi-square test was used for tests of association. Mann-Whitney nonparametric tests were used to compare the time to revision surgery, as this data did not have a normal distribution. Odds ratios were calculated from 2 × 2 contingency tables.

Statistical significance was defined as a *p* value ≤0.05 with a 2-sided hypothesis.

## 3. Results

During the study period, 109 patients were enrolled, from a total of 202 patients who underwent hip and knee joint revision surgery involving the exchange of at least one component. We excluded 3 patients for breach of protocol, because their larger implants were sent to the lab in plastic bags. Sonication of implants in plastic bags has been shown to have a high risk of contamination and therefore false positive results [7].

The final study population was 106 joints from 106 patients. Fifty-four joints were revised for aseptic loosening of the implant (aseptic study group), and fifty-two joints underwent revision for other reasons (control revision group) such as isolated polyethylene wear (16/106), dislocation (12/106), periprosthetic fracture (8/106), and finally mechanical failure (16/106).

There were no significant differences between our groups in the type of joint revised (*p* = 0.51) or the indication for index arthroplasty (*p* = 0.832). Furthermore, there was no difference in the patients' age at either index or revision surgery (*p* = 0.274 and *p* = 0.452). Finally there was no difference in the use of cement between the groups (*p* = 0.073). There was a significant difference (*p* = 0.033) in the gender distribution between the groups with females overrepresented in the control revision group (*p* = 0.033) and those patients with a history of smoking (*p* = 0.007). There was no significant difference in the lifespan of the implant prior to revision between aseptic loosening (14 years) and control revision (10.3 years) groups (*p* = 0.054) (Table 1).

There were 9 positive cultures in the study, eight (15%) positive cultures in the aseptic loosening group and 1 (2%) in our control revision group (*p* = 0.017, associated odds ratio of 47.7). Any positive culture on swab, aspirate, or deep tissue was taken to represent a positive culture on conventional sampling technique. These nine patients had an average post-op documented clinic follow-up in the medical records of 9.7 months (range 3–22 months) and 19.3 months (range 8–31 months) of survivorship at writing of the manuscript without known representation. Four patients were treated in consultation with a specialist infectious diseases team with oral and/or intravenous antibiotics. All patients underwent a single stage revision and none have had any ongoing complications, repeat admissions, or operations due to infection of their prosthesis.

Out of the 8 cases in the aseptic loosening group with positive conventional cultures, 3 cases had positive cultures from a single sampling technique. Those cultures were from deep tissue samples, where multiple specimens from different regions around the joint undergoing revision were positive with the same organism.

We found concordant results between sonication fluid culture and conventional samples in 5 of the 9 cultures (56%). Sonicated fluid culture was positive in one case where conventional cultures were negative. Three patients from the aseptic loosening group had concordant growths from both conventional and sonication cultures (Table 3). We found more cultures to be positive on conventional sampling alone than on sonicated fluid culture alone (4 compared to 1). Conventional sampling techniques provided more positive cultures than sonication. Examination of the conventional sampling techniques indicated that deep tissue sampling was the most likely to be positive with synovial fluid aspirates the least likely to be positive (Table 4).

The microorganisms cultured were mainly* Staphylococci* with coagulase-negative* Staphylococci* (CONS) the most commonly cultured organisms in the aseptic loosening group and the only species cultured from the only positive control case.* S. aureus* was the other cultured organism, being positive in 2 cultures from the aseptic loosening group.* Staphylococci* were the only microorganisms to be cultured from the sonicated fluid samples in this study (Table 2).

We compared the results of those who returned a positive culture with the previously recorded risk factors and found that none of these factors were associated with an increased risk of returning a positive culture. There was also no statistically significant association between having raised or abnormal preoperative neutrophil differential counts, CRP or ESR, and having a positive culture result (Table 5). There were three patients in the aseptic loosening group and four patients in the control revision group who had an isolated elevated preoperative CRP. In the aseptic loosening group, two of the three patients had a CRP within 2 mg/L of the cutoff point of 10 mg/L. There were no other features to suggest infection at the time of workup. One of these patients returned positive cultures of CONS at revision from all methods and was treated as an occult infection. The third patient had a negative preoperative radiologically guided aspirate for a significantly elevated CRP and hence was included in the aseptic loosening group. Of the four in the control group, one had a CRP within 1 mg of the cutoff point, two had a background of rheumatoid arthritis and were receiving steroids, and one had a periprosthetic fracture and concomitant lower limb cellulitis at the time of admission. None of these patients had positive cultures from the revision procedure.

## 4. Discussion

Although we found a significant positive culture rate from patients undergoing revision for presumed aseptic loosening, our study did not support the sonication of retrieved implants at revision surgery for aseptic loosening despite the literature suggesting that implant sonication is a more sensitive technique for the detection of infection. We consistently had more positive cultures from conventional sampling methods combined than on sonication. This reduced sensitivity of detection is out of keeping with the published literature around the use of sonication [6–10]. Our implants were placed into a sterile sealed solid container as opposed to sealed plastic bags given the concerns about contamination. Trampuz et al. found that there was clear evidence of bag leakage after sonication and showed reduced specificity with some growths of water related bacterial organisms [11]. To our knowledge, most other sonication protocols looked at sonication in bags which therefore could be subject to the same issues of contamination. Our methodology aimed to obviate that risk; however, it may be that the solid container decreased the efficacy of energy transmission and therefore cavitation between the sonicated fluid and metal interface, leading to our seemingly reduced sensitivity as compared with conventional sampling techniques. As part of the study design, we undertook a viability study to ensure that the sonication and culturing process was not compromising microorganism viability. Nevertheless, our* in vitro* assessment did not directly assess the ability of our sonication protocol to work on implants in a solid container.

To our knowledge, this study is the first to use a control revision arthroplasty group for comparison when assessing the rate of infection in revision for aseptic loosening whereas prior literature has focused on the aseptic loosening and infection groups exclusively. Our study groups were very similar despite being an unmatched prospective case-control series. The gender discrepancy between groups could relate to higher proportion of female patients who underwent revision for periprosthetic fracture and dislocation. Nguyen et al. studied the role of sonication in 21 femoral component revisions from both hip and knee replacement surgeries for aseptic loosening [8]. They had an intraoperative control implant opened at the time of surgery and processed concurrently to validate their sonication protocol. They had stringent criteria for excluding cases from their aseptic group such as any derangement in preoperative inflammatory markers. They found 4.7% (1/21) positive growth from sonicated fluid in this group of patients but also had one case of contamination.

We found a 15% positive culture rate in our presumed aseptic loosening group. This was significantly higher than the recent literature that has directly or indirectly examined this problem [7, 8, 11, 12], but well below earlier work by Tunney et al. [10, 13]. Tunney et al. had a 10% positive sonicated culture rate in 120 implants and 73% PCR positive rate (mainly* Staphylococcus* and* Propionibacterium* species). However, in their work, Tunney et al. did not screen for clinical infection. Trampuz et al. documented a 9% culture rate from conventional sampling in a series of 54 patients [9], but later research demonstrated a much lower rate of 2% in a larger series of 252 revisions for aseptic loosening [11].

By comparing the aseptic loosening group to those undergoing revision for isolated polyethylene wear, dislocation, and fracture, respectively, without implant loosening, the results clearly show that loosening is associated with higher rates of occult infective process. Whether the higher rates of bacterial growth are indicative of occult infection being the cause of loosening or whether loosening creates a favourable environment for bacterial growth is difficult to determine. Regardless, according to the current Musculoskeletal Infection Society definition, the isolation of the same pathogen from two separate tissue samples obtained from a prosthetic joint constitutes a PJI and therefore the patients would receive treatment [14]. Coagulase-negative* Staphylococcus* was our most commonly cultured organism. Because of the multiple processes used to validate our methodology, we are confident that our results are not false positives (contamination).

Despite having incomplete preoperative inflammatory marker testing, we have also shown that there still remain a significant number of patients who are likely to have occult infection at revision for aseptic loosening despite normal preoperative ESR and CRP. The best method to confirm or exclude infection was deep tissue samples at the time of surgery. We had three patients in the aseptic loosening group with an elevated CRP preoperatively, but only one of them had a positive culture from deep tissues. Conversely, four patients in the control group had elevated inflammatory markers preoperatively and none of them had evidence of occult infection. Our results indicate that a negative preoperative workup with inflammatory markers should not replace adequate microbiological sampling at the time of revision surgery especially for cases of aseptic loosening as compared to other causes of revision. One should use inflammatory markers to stratify the risk of infection in their patients before proceeding with revision surgery as per international guidelines [2].

The rate of clinical infection developing in cases with unexpected positive culture results at the time of revision surgery in the literature has varied from 0 to 11% depending on the length of follow-up and on the initiation of therapeutic antibiotics after positive results [10, 12, 13]. Our approach has been to treat any deep tissue cultures which are multiply positive with appropriate antibiotic therapy in consultation with an infectious diseases team as this meets the current diagnosis of PJI and we felt that the benefits outweighed the potential downsides of treatment with antibiotics. We consider the possibility of the positive result being a contaminant is unlikely in this study because of our control group, and the quality control tests, which validated our methodology.

There are several limitations to our study, for example, the variability of use of prophylactic antibiotics on induction of anaesthesia. However, we were unable to control this as it was a restriction required to get ethical approval for the study. Furthermore, given the premise that bacteria in biofilms are more resistant to antibiotic administration, this would, if anything, underestimate the prevalence of occult infection in the aseptic loosening group. This would add further credence to the utility of microbiological sampling at the time of revision for patients with loose implants.

A further methodological weakness includes an incomplete set of inflammatory markers for all patients undergoing revision. Within that context, however, we were able to find significant* Staphylococcus* growths in patients who had negative preoperative inflammatory markers. Given our study design, we can not comment on the specificity or sensitivity of the preoperative blood workup on the diagnosis of PJI other than to say that negative preoperative assessment does not always indicate lack of infection or the converse.

Another limitation was the lack of histopathological review of tissues in our study. A comparison between the positive culture patients and their routine microscopic histological assessment would have been beneficial as added evidence of the presence of infection. Previous literature however has shown that deep tissue culture when positive is highly specific for PJI [15, 16]. We did not undertake frozen section intraoperatively. Tsaras et al. showed that the presence of 5 neutrophils (PMN) in five high power fields (HPF, 400x magnification) increased the likelihood of infection 12-fold [17]. Furthermore, they showed that there was no significant difference between 5 and 10 PMN/HPF cutoffs. However, it is our experience that frozen section is highly subjective and its utility in PJI sampling is unpredictable. In their study, Tunney et al. found that histopathology could only be suitably assessed on 67.5% (81/120) of their cases [10]. Based on a cutoff of >10 PMN/HPF, only 4/26 patients with positive cultures had an abnormal frozen section suggestive of infection. Furthermore, 7/94 culture negative cases had frozen section features to suggest infection but no positive cultures despite their increased sensitivity using sonication. We therefore feel that our results are valid despite the lack of histopathological comparison.

Finally, we did not undertake PCR analysis on the sonicated fluid cultures in our study. PCR of the 16S rRNA segment of the bacterial genome offers a highly sensitive assessment for the presence of bacteria. Ince et al. found one positive culture on PCR of tissue samples in a series of 24 revision procedures for aseptic loosening, but they did not assess the role of sonication [6]. Tunney et al. had an overall 72% positive rate of PCR in their study of sonicated fluid culture [13]. This comprised a 100% PCR positive rate in their culture positive group and 40% rate in their culture negative group. This led them to assume that occult infection is present at a much higher rate than previously thought. However, they only excluded skin contamination from the staff at the laboratory undertaking the analysis and not the operating room personnel or environment. Kobayashi et al. undertook an assessment of ultrasonication and real time intraoperative PCR in 23 patients undergoing 30 reoperations of a large joint prosthesis [18]. They do not give a clear indication of the reasons for reoperation but the sample included operations for the treatment of infection. They found that intraoperative PCR was positive in 13/15 patients who had conventional positive cultures. The sensitivity and specificity of their sonicated fluid PCR were 0.8 and 0.87, respectively, when compared to conventional cultures and histopathology combined.

The presence of bacterial 16S rRNA material on PCR does not equate with the presence of active or indolent bacterial infection. It merely indicates the presence of bacterial genetic material rather than bacterial material viability or pathogenicity. The test is also highly sensitive, which makes any potential contamination more likely to be detected. The 16S rRNA segment is very common to most bacteria known to cause PJI; therefore, its use is reportedly associated with increased sensitivity [19]. PCR targeted at organisms such as Methicillin Resistant* Staphylococcus aureus* was more specific but lacked sensitivity [18]. In a meta-analysis of 14 articles looking at PCR, Qu et al. found that PCR had a sensitivity of 0.86 and specificity of 0.91 for PJI [19]. They also noted a heterogeneity of the study materials and that quantitative PCR was more accurate compared to nonquantitative assessments. Currently, the role of PCR is not part of the standard definition of PJI but may be a useful adjunct in the management of culture negative overt PJI.

## 5. Conclusion

We found a positive culture from intraoperative sampling in patients undergoing revision for aseptic loosening group of 15%, and as such previously unrecognised infection was present in a clinically significant proportion of this group. Ultrasound sonication of the removed prosthesis was less sensitive than conventional sampling techniques in our study. This could be due to ultrasonication in solid containers as opposed to bags to reduce contamination risk. We suggest that unrecognised infection may be present in a significant proportion of aseptic loosening cases undergoing revision surgery and that routine intraoperative sampling is recommended, but our results do not support the routine use of ultrasound sonication for its detection. The question remains whether loosening is caused by low grade infection or whether infection is promoted in the environment of loosening.

## Figures and Tables

**Table 1:** Comparison between the study groups (comparison by Chi-square test and ANOVA means comparison).

Factor	Aseptic loosening *n* = 54	Control *n* = 52	*p* value	Odds ratio
Male sex	33 (61%)	21 (40%)	0.033	
Mean age at index arthroplasty (yrs)	57.4	60.4	0.274	
Mean age at revision arthroplasty (yrs)	72.3	70.7	0.452	
Mean number of years since index operation (95% CI)	14.0 (11.2–16.8)	10.3 (8.1–12.5)	0.054	
Joint type				
Hip	37 (70%)	40 (75%)	0.51	
Knee	16 (30%)	13 (25%)		
Cemented implants	25 (46%)	22 (42%)	0.073	
Smoking history	7 (13%)	1 (2%)	0.007	7.6
Indication for index replacement				
Degenerative	45 (83%)	42 (81%)	0.823	
Inflammatory arthritis	3 (6%)	3 (6%)		
Posttraumatic arthritis	4 (7%)	3 (6%)		
Other	2 (4%)	4 (8%)		

**Table 2:** Positive culture case series.

Patient number	Aseptic loosening (AL) or control (C)	Joint involved	Conventional culture	Sonication culture	Organism	Treatment	Clinical follow-up	Months since index procedure	Infection related complication
2	A.L	Uncemented THA	N	Y	*S. aureus *	Oral Abs.	22	31	No
7	A.L	Hybrid THA	Y	N	CONS	NIL	3	27	No
18	A.L	Cemented THA	Y	N	CONS	NIL	15	24	No
19	A.L	Uncemented THA	Y	Y	CONS	ID/Abs.	5	23	No
32	C	Uncemented THA	Y	Y	CONS	NIL	10	13	No
70	A.L	Uncemented THA	Y	N	CONS	NIL	12	18	No
96	A.L	Cemented THA	Y	Y	CONS	ID/Abs.	3	13	No
107	A.L	TKA	Y	N	*S. aureus *	ID/Abs.	8	9	No
110	A.L	TKA	Y	N	CONS	NIL	6	8	No

**Table 3:** Breakdown of positive cultures into sampling technique.

Study group	Conventional only	Sonication and conventional	Conventional	Total (%)	Odds ratio	*p* value
Aseptic loosening (*n* = 54)	4	1	3	8 (15%)	7.7	0.017
Control revision (*n* = 52)	0	0	1	1 (2%)		

**Table 4:** Frequency of sampling method returning a positive culture.

Study group	Aspirate	Swab	Tissue	Conventional combined	Ultrasonication
Aseptic loosening	2	3	5	7	4
Control revision	1	1	1	1	1

**Table 5:** Preoperative blood tests (Chi-Square test).

Blood test		Any positive cultures		Totals	*p* values
		Y	N		
CRP (normal <10 mg/L)	Normal	6	43	49	0.89
	High	1	6	7	

ESR (normal 1–20 mm/hr)	Normal	8	46	54	1
	High	0	0	0	

WCC (normal 4–11 × 10^9^/L)	Normal	7	41	48	0.181
	Abnormal	1	1	2	

Neutrophils (normal 1.7–7.5 × 10^9^/L)	Normal	7	41	48	0.407
	Abnormal	1	2	3	
